# Euthanasia tactics: patterns of injustice and outrage

**DOI:** 10.1186/2193-1801-2-256

**Published:** 2013-06-06

**Authors:** Brian Martin

**Affiliations:** Law, Humanities and the Arts, University of Wollongong, Wollongong, NSW 2522 Australia

**Keywords:** Euthanasia, Injustice, T4 programme, Tactics, Death with dignity

## Abstract

Struggles over euthanasia can be examined in terms of tactics used by players on each side of the issue to reduce outrage from actions potentially perceived as unjust. From one perspective, the key injustice is euthanasia itself, especially when the person or relatives oppose death. From a different perspective, the key injustice is denial of euthanasia, seen as a person's right to die. Five types of methods are commonly used to reduce outrage from something potentially seen as unjust: covering up the action; devaluing the target; reinterpreting the action, including using lying, minimising consequences, blaming others and benign framing; using official channels to give an appearance of justice; and using intimidation. Case studies considered include the Nazi T4 programme, euthanasia in contemporary jurisdictions in which it is legal, and censorship of Exit International by the Australian government. By examining euthanasia struggles for evidence of the five types of tactics, it is possible to judge whether one or both sides use tactics characteristic of perpetrators of injustice. This analysis provides a framework for examining tactics used in controversial health issues.

## Background

Decades ago, most people died as a result of disease that ran its course, without significant medical or technological interventions. Before hospitals, people were most likely to die at home, in what was often seen as a natural process.

This common experience of death has changed in industrialised countries, especially since the 1950s. Medical technology, for example transfusions, respirators and feeding tubes, in conjunction with medical and legal systems, has enabled the extension of life far beyond what was possible in earlier times. Many people now die in hospitals, often after intensive medical care that lasts for days, months or years. Death is less commonly experienced as a natural process and more frequently as the end-point of medical interventions to maintain life.

End-of-life care includes medical treatment for those with a prospect of recovery and palliative care to ease suffering. Medical professionals recommend or sometimes decide the mode of care: in this sense, most death is medicalised to some degree (Clark 2002).

Artificial extension of life, along with medicalisation, has undoubtedly benefited many individuals who are able to live much longer and pain-free than in previous eras, and to gain satisfaction from their increased longevity. However, there is also a negative side to this process: some people experience the period before their death as painful, distressing or humiliating — so much so that they would prefer to die sooner rather than later. For them, technology has enabled or even forced an extension of suffering. These experiences of suffering, and witnessing loved ones suffer in their final time alive, have increased support for access to euthanasia, interpreted as a peaceful death at a time and place of one’s choosing.

Partisans in the contentious debate over euthanasia use different terms, and there is no agreement among writers in the area (Gailey 2003;pp. 133–138; Somerville , pp. 133–138; Somerville pp. 133–138; Somerville 2001). For convenience, I use the word “euthanasia” to include the three categories of dying most frequently used in discussions of the topic. In the first, commonly called “physician-assisted suicide” or PAS, a physician provides the means, such as drugs, that enable a person to end their own life. The second type involves a physician taking the initiative, such as giving a lethal injection (often called “euthanasia” or “active euthanasia” as distinct from PAS). In the third type, individuals achieve their own desired peaceful death without assistance from others, an act sometimes called “self-deliverance”.

Most of the public debate (ProCon.org 2013) and voluminous writing (Yount 2007) on euthanasia approaches it from medical, legal or ethical angles. For example, palliative care doctors and nurses may differ about informed consent or about how effective palliative care can be. When their efforts to reduce suffering hasten death, this is justified through the principle of double effect: as long as the intention is to reduce suffering, interventions are justified (Sulmasy and Pellegrino 1999).

Legislators and courts deal with the legal regulation of end-of-life treatment. In most countries, PAS and active euthanasia are illegal. In a few places — Netherlands, Belgium, Colombia, Luxembourg, Switzerland and the US states of Oregon, Washington and Montana — one or both are legal or permitted in practice (Côté 2012). Opponents argue against existing and further legalisation, seeing it as a slippery slope to abuses including killing members of vulnerable groups such as the elderly and people with disabilities (e.g., Smith 1997). One of their arguments is that improved palliative care should make euthanasia unnecessary (Jeffrey 2009).

A key ethical issue is whether measures taken to hasten death are ever justifiable. Philosophers have examined this in great detail (Battin 2005). Campaigners on both sides of the debate have used ethical claims as rhetorical tools. It is predictable that campaigners present their own stances as ethical and their opponents’ stances as unethical.

I approach this contentious issue from a new angle: an examination of tactics used by key players to reduce outrage from ethical violations. The key question is, what do those who assist others to die do to reduce potential outrage over their actions?

In the next section, I introduce a model for examining tactics used by perpetrators of what others potentially perceive as unjust. Following this, I examine euthanasia — especially without consent — as an injustice, giving examples of methods used to reduce outrage and ways to counter these methods. A key case study is the infamous Nazi T4 programme. Then I provide a parallel examination of the *denial* of euthanasia as an injustice, again giving examples of methods of reducing outrage and ways to counter these methods. In the conclusion, I sum up the implications of this approach for studying the euthanasia debate and for campaigners who want to oppose one or both injustices involved.

## Outrage management

Few social scientists have made strategy and tactics, as deployed in controversial issues, the focus of attention. Goffman ( 1970) examined strategic choices in everyday interpersonal behaviour but did not address strategy in public debates. Various authors (e.g., Gorsevski 2004;Jowett and O’Donnell 2006;Mathiesen 2004;Richardson et al. 1993) have addressed strategy in the realm of discourse (mostly fitting into the category of reinterpretation, discussed below), but few have looked beyond this domain, for example at actions of police and courts. James Jasper, in his treatment of everyday strategic dilemmas, commented, “My research on social movements showed me just how little social scientists have to say about strategy” (Jasper 2006, p. xii). Jasper’s goal was to highlight the complexities of strategic interaction, whereas my purpose here is to examine regular patterns. For this, I turn to studies of outrage against injustice.

Social historian Barrington Moore, Jr. ( 1978) observed that in every society there is a sense of justice or fairness. Some actions are condemned as unfair, for example one person hitting another without any pretext. Although some ideas about what is unjust are found in nearly every society, others develop in particular times and places. For example, in many places centuries ago, public executions were commonplace and popularly attended, a practice that many people today would find abhorrent.

Whenever an action is seen as unjust, unfair, unethical or otherwise inappropriate, perpetrators risk generating an adverse popular reaction that can go by labels such as outrage, anger, concern, disgust or revulsion. To prevent or reduce such reactions, perpetrators can adopt various tactics. These can be classified into five categories of outrage management (Martin 2007):

- cover-up of the action.
- devaluation of the target.
- reinterpretation of the events, including by lying, minimising, blaming and framing.
- using official channels to give an appearance of justice.
- intimidation.

As an example, consider torture. Today it is almost universally condemned, in part due to the efforts of human rights movements. Though every government in the world today officially rejects torture, it is widely practised (Boltzmann Institute of Human Rights 2013). Therefore, torturers typically use the following sorts of outrage-management tactics.

- *Cover-up* Torture is invariably carried out in secret.
- *Devaluation* The victims of torture are denigrated, for example as terrorists, subversives or criminals.
- *Reinterpretation* When allegations of torture are made, perpetrators often lie about the occurrence, scale or identity of victims. They may minimise the harm caused by certain forms of interrogation, such as waterboarding. Senior officials may blame rogue operators. They may say that what occurred is not really torture and use terms like “abuse”.
- *Official channels* When allegations of torture receive public attention, governments sometimes establish inquiries that, through their lengthy and procedural mechanisms, allow public concern to fade.
- *Intimidation* Victims of torture are commonly threatened with reprisals to themselves or their families if they speak out. Some of those who expose torture, for example whistleblowers or journalists, are threatened.

Every one of these techniques was used in relation to torture at Abu Ghraib prison in Iraq (Gray and Martin 2007).

Though the characteristic techniques vary from issue to issue, the same techniques can be observed in diverse injustices, for example censorship (Yecies 2008), defamation (Gray and Martin 2006), sexual harassment (McDonald et al. 2010), corporate disasters (Engel and Martin 2006), treatment of refugees (Herd 2006) and genocide (Martin 2009). Therefore, it seems plausible to look for these methods of reducing outrage in the case of euthanasia.

Note that in many issues there are competing injustices or competing ethical considerations. In the case of torture, for example, the torture itself can be seen as cruel, but the competing injustice is failure to extract timely information from the person tortured — if indeed that is the point of the torture. The analysis of tactics can apply in either direction, but typically one side has far more power than the other — a torturer has vastly more power over the person tortured — and powerful perpetrators usually have a greater capacity to use techniques to reduce outrage, especially official channels and intimidation.

It is also worth noting that different people react differently to any given event. To speak of public outrage from an action implies outrage by some significant proportion of the population. For example, the My Lai massacre — killing of hundreds of Vietnamese civilians by US troops in 1968 — was seen as many as despicable, but significant numbers of US soldiers and civilians saw it as justifiable or even laudable (Kelman and Lawrence 1972;Opton 1972). Nevertheless, the likelihood of adverse reactions was significant enough for soldiers, commanders and politicians to use all five methods of reducing outrage (Gray and Martin 2008).

This same divergence of responses is a characteristic feature of the euthanasia debate, which has evoked strong emotional responses for decades. To apply the outrage-management framework, the basic approach is to examine the actions taken that might prevent or reduce outrage from an action potentially seen as unjust. This can be done when there is a divergence of responses to an action, but with the extra dimension of looking at tactics used in relation to both euthanasia and its denial.

## Euthanasia as an injustice: tactics and counter-tactics

When many people see euthanasia as bad — for example, as a denial of the right to life or simply as murder — then it is to be expected that perpetrators will take steps to reduce adverse reactions.

Consider first the notorious Nazi T4 programme, commonly called the euthanasia programme, seen as so outrageous that it stigmatised all forms of euthanasia for decades. The Nazi programme of killing people with disabilities was authorised by Hitler in 1939 and officially halted in 1941. Unknown to most, the children’s killing programme was exempted from the halt decree; furthermore, killings continued locally until the end of World War II.

Some might say that to refer to the T4 programme as euthanasia is completely inappropriate: there was no compassionate intention as the Nazis tried to suggest by using the euphemisms “euthanasia” and “mercy death” (Friedlander 1995, p. xxi), but simply a programme of killing given a deceptive patina of legitimacy. Whatever one’s assessment of the Nazi programme, it can be analysed in terms of tactics used by the perpetrators to reduce outrage. It is worthwhile to learn from the tactics in the Nazi case in order to set up and assess responsible euthanasia processes, namely ones that do not rely on the same sorts of tactics.

### Cover-up

The Nazi programme was not publicly announced or explained. Quite the contrary: “Immense pains were taken to keep T–4’s operations covert” (Burleigh 1994, p. 162). To run the programme, the obscure agency KdF (Chancellery of the Führer) was chosen because of its small size and low visibility (Friedlander 1995, p. 40).

Though the programme was classified top secret, many knew about it, notably doctors involved. They were perpetrators and aided in the cover-up. They hid their actions from those most likely to be disturbed by and protest against the killings, including relatives, members of the Catholic Church, and foreign populations (Aly 1994, pp. 29–32). After public protest led to the closing of two killing centres, transit institutions were created to add greater secrecy to the process (Friedlander 1995, p. 108).

### Devaluation

Under Nazi rule, people with disabilities were commonly labelled “idiots,” “crazies” and “cripples.” Perpetrators used the expression “life unworthy of life” (Friedlander 1995, p. xxii). The eugenics movement, strong in Germany as well as some other countries, devalued anyone deemed to have defective genes (Proctor 1988). The Nazis produced propaganda films devaluing people with disabilities (Burleigh 1994, pp. 183–219). For example, the 1936 film Erbkrank was intended “to criminalise, degrade and dehumanise the mentally and physically handicapped so as to justify compulsorily sterilising them” (Burleigh 1994, p. 183). Devaluation, at a psychological level (Bandura 1986, pp. 375–389), also helped enable the killings.

### Reinterpretation

Language is the most obvious part of reinterpretation: as noted, the Nazis used the terms “euthanasia” and “mercy death” to describe the killings, which otherwise would be called murder. Another reinterpretation technique was to rationalise killings by saying that people in institutions were expensive drains on the Nazi state when facilities were needed for injured soldiers.

Outright lying was another standard reinterpretation technique. For example, patients from institutions were transferred to other centres for killing, with guards in white coats in attendance to make it seem like a medically supervised process. Parents were told that their children were being sent to special centres where they would receive better treatment. Relatives were sent death certificates with false information about the cause of death (Burleigh 1994;Friedlander 1995, pp. 85, 98–106). To disguise the central direction of the programme, physicians and administrators used pseudonyms (Friedlander 1995, p. 103). Lying can serve as a form of cover-up; it fits within the category of reinterpretation when relatives knew that something had happened — death of a loved one — but were deceived about how and when it occurred.

### Official channels

The programme was never given legal approval; Hitler refused this because the German people would not support it (Friedlander 1995, p. 154). Instead, Hitler wrote a letter privately authorising the programme and this letter was used to win over some participants (Burleigh 1994;pp. 112–113; Friedlander , pp. 112–113; Friedlander pp. 112–113; Friedlander 1995, pp. 67, 154). A formal meeting served to win over sceptical legal professionals (Burleigh 1994;pp. 172–173; Friedlander , pp. 172–173; Friedlander pp. 172–173; Friedlander 1995, p. 122).

In August 1941, Hitler halted the programme, but this official response to protest was deceptive. The “halt” only applied to killing centres and did not apply to children. T4 continued, with physicians killing adults using starvation and injections (Burleigh 1994;Friedlander p. 180; Friedlander 1995, p. 154).

It was only after the war that official channels, namely courts, were used against the perpetrators. After 1947, the German Federal Republic judiciary mostly made decisions that allowed T4 participants to rejoin German professions, for example by terminating trials, acquitting defendants or giving lenient sentences (Bryant 2005, p. 218).

### Intimidation

Speaking out against Nazi policies was always risky. Parents who refused permission for their children with disabilities to be sent away were threatened with being sent to work camps or having all their children taken into state custody (Burleigh 1994;Friedlander p. 166; Friedlander 1995, pp. 59–60). After Bishop Galen’s sermon condemning the T4 programme (discussed later), ordinary Germans found to possess, circulate or discuss the sermon were subject to reprisals including losing jobs, being sent to concentration camps or execution (Burleigh 1994, pp. 178–180).

The perpetrators of the Nazi T4 programme thus relied on all five types of tactics to reduce outrage — a very strong indication of the potential for popular outrage about the programme. This is exactly what is to be expected using this analysis of tactics: when powerful perpetrators anticipate resistance, they are likely to use a range of tactics that reduce outrage.

What then about challenging the programme? Methods of doing this can be categorised into five types of counter-tactics to the five types of outrage-reduction tactics.

### Exposure

The key to challenging cover-up is to get information to receptive audiences. Information about the T4 programme gradually leaked out via observations and inferences by relatives and local people (Burleigh 1994;pp. 162–164; Friedlander , pp. 162–164; Friedlander pp. 162–164; Friedlander 1995, p. 111). The breakthrough event was a 1941 pastoral letter by Clemens August von Galen, bishop of Münster, which was reprinted and widely distributed throughout Germany (Burleigh 1994;Friedlander p. 178; Friedlander 1995, p. 115).

### Validation

To challenge devaluation, the victims needed to be conceived as humans with lives worth living. Von Galen ( 1941), referring to the targets of the T4 programme, said “we are dealing with human beings, with our neighbours, brothers and sisters,” describing them positively in terms of vital relationships. (Incidentally, von Galen was far less vocal about the value of Jewish lives [Griech-Polelle 2001).

### Reframing

To counter techniques of lying, minimising, blaming and framing, the programme had to be named as an injustice, namely killing pure and simple. One asylum director, Heinrich Hermann, used the word “killing” in criticising T4 to visiting euthanasia planners, who were disconcerted by the direct language: “These institutional murderers with civil servant status were not equal to a confrontation with the naked word ‘killing’” (Aly 1994, p. 34).

### Mobilisation

There are two ways to respond to official channels used to give a deceptive appearance of justice. One is to avoid or discredit the official channels. In the long term, discrediting the Nazi regime accomplished this, so much so that virtually any Nazi policy was discredited by association. The other response is to not rely on official channels for redress but instead to mobilise support among the public, for example by talking to individuals, publicising the issues, holding private or public meetings, forming networks and groups, and making public protests.

For many months prior to von Galen’s pastoral letter, various individual opponents of T4 — especially church people — wrote letters to or had meetings with government officials, such as the Ministry of Justice, but this insider approach achieved little (Burleigh 1994;pp. 166–176; Friedlander , pp. 166–176; Friedlander pp. 166–176; Friedlander 1995, pp. 113–114, 121–122). These were significant signs of opposition but they were not so effective as mobilising public support: they essentially relied on an official channel, namely appealing to government officials, that gave only the appearance of offering a solution.

### Resistance

The counter to intimidation is to continue taking action against the injustice and to expose evidence of intimidation in order to create greater outrage. Those who opposed the euthanasia programme at the time, in word or deed, displayed incredible courage (Gallagher 1995, pp. 137–146, 186–203).

In summary, the Nazi T4 programme is an ideal illustration of an injustice in which all the methods of reducing and fostering outrage can be observed.

In the current debate over euthanasia, the issues are not as clear-cut as in the Nazi case. Nevertheless, the same sorts of methods are involved. As a typical example, consider the case of a patient, with a terminal illness, whose doctor believes is suffering unnecessarily and would be better off dead. Suppose the doctor, without informing or obtaining permission from anyone, gives this patient a lethal drug dose. This can occur whether or not active euthanasia is legal (Kuhse et al. 1996;Magnusson 2002).

The primary means of preventing outrage is cover-up: the doctor tells no one about intentionally hastening death. Other doctors or perhaps relatives might suspect what the doctor has done, but not have sufficient evidence or incentive to expose it. If there is a suspicion of foul play, then the doctor may use reinterpretation, saying the person was going down quickly or claiming not to have been involved. In such situations, devaluation may or may not be used. The doctor seldom has access to official channels to reduce outrage, nor has any means of intimidating critics.

This assessment shows that covert euthanasia by individual doctors is quite different — in terms of available methods for reducing outrage — to the Nazi T4 programme. The primary techniques available to individual doctors are cover-up and reinterpretation. Without the backing of a powerful institution, such as the Nazi state, other methods of reducing outrage are unavailable or of limited assistance.

In practice, covert euthanasia of this sort ranges from sincere efforts to minimise suffering done on an ad hoc basis to regular killing that is more readily labelled criminal, as in the case of the British serial-killer doctor Harold Shipman (Whittle and Richie 2004).

When euthanasia has been legalised, a different pattern emerges. Following a set of legal guidelines, physicians may assist a person to end their life (as in Oregon) or administer a lethal injection (as in the Netherlands). Consider an instance of legal euthanasia in which the patient has a terminal illness, is suffering, requests death and is provided with lethal drugs that can be self-administered.

### Cover-up

There is no cover-up: the person dying knows quite well that the purpose of taking the drugs is to cause death. In some cases relatives and friends are present.

### Devaluation

Whether devaluation is involved is a matter of debate. Critics of euthanasia might say the person’s life, by being shortened, has been devalued by that fact; supporters might say that euthanasia actually involves respect for a person’s autonomy and for their experience of life, which has become intolerable.

### Reinterpretation

Those involved with legal euthanasia do not lie about what is happening: they are obliged to open about it. There is no evidence that they minimise the significance of what they are doing; many find it distressing (e.g., Kade 2000). They cannot blame someone else for the death: they have to accept responsibility. The most important reinterpretation technique used is framing: they say that what occurs is voluntary and intended to reduce suffering — not an unfair termination of life and certainly not killing. For example, the Dutch government brochure Euthanasia: The Netherlands’ New Rules defines euthanasia as “the termination of life by a doctor at the patient’s request, with the aim of putting an end to unbearable suffering with no prospect of improvement” ( Ministerie van Volksgezondheid, Welzijn en Sport, n.d.).

Some would say that the Oregon state model is less than fully open, in that deaths in which physicians provide assistance are officially called natural deaths. The Swiss model is more transparent: legal oversight is provided by the criminal justice system; police investigate every death for legal compliance; and some right-to-die societies film deaths and immediately provide videotapes to the police (Ziegler 2009). This also allows research into euthanasia deaths (Ogden et al. 2010) and thus greater understanding.

### Official channels

When proper procedures are followed, official channels authorise the process of dying. Critics of euthanasia allege that consent sometimes has not been given or that cases are not reported as they are supposed to be (Jeffrey 2009;pp. 63–73, 84; Smith , pp. 63–73, 84; Smith pp. 63–73, 84; Smith 1997, pp. 90–114). However, it is very difficult for critics to use complaint procedures to challenge decisions, because the laws authorising euthanasia provide protection to doctors and others involved if formal procedures are followed.

### Intimidation

There is little evidence that critics have been threatened or penalised. (See, for example, Thomasma et al. 1998).

In summary, legal euthanasia involves few of the techniques typically used by powerful perpetrators to reduce outrage from injustice. There is little or no cover-up, little devaluation, reinterpretation only by framing, and little intimidation. The one exception is official channels: laws and judicial practice provide powerful protection for doctors and others involved in the process.

Note that this analysis does not by itself provide an endorsement of legal euthanasia. All it shows is an absence of most of the techniques commonly used by powerful perpetrators of actions potentially seen as unjust.

In this section, I have outlined the common tactics used by perpetrators to reduce outrage over euthanasia and some of the counter-tactics that can be used by those opposed to euthanasia. The Nazis, in killing hundreds of thousands of people with disabilities, used the full range of techniques for reducing outrage: cover-up, devaluation, reinterpretation, official channels and intimidation. On the other hand, individual doctors who practise euthanasia, where it is illegal, typically rely primarily on cover-up. When euthanasia is legal, the pattern is quite different: only official channels might be seen as tools to reduce outrage.

So far I have analysed euthanasia in terms of tactics used to reduce concern. A different analysis, to which I now turn, is of tactics used to reduce concern about the opposite injustice: refusal of euthanasia.

## Denial of euthanasia as an injustice: tactics and counter-tactics

Many people believe that a person in great suffering, with little chance of relief, and who wants to die, should be allowed to do so (ProCon.org 2013). To deny such a death can, to them, be seen as inhumane, and they may be disturbed, concerned, upset or outraged. Because of this possible reaction, those who prevent such deaths may act to reduce adverse reactions. My aim here is to look at the tactics used for this purpose. Given that tactics can vary from country to country and time to time, I take some examples from Australia in recent years, for which the evidence is particularly striking.

### Cover-up

The first type of cover-up concerns the suffering that many individuals experience in being denied a desired peaceful death, including pain, discomfort and indignity. Information about such experiences is not censored officially, but is seldom publicised by doctors or relatives.

The second type of cover-up is about ways to achieve a peaceful death. This sort of information is not commonly publicised. It is relatively easy to learn about death from violent means, because of news reporting and television shows showing murders and suicides, but there are relatively few detailed descriptions of desired deaths using drugs, exit bags or other methods (for examples, see Ogden 2010a, b).

The Australian federal government has taken cover-up to another level by passing a law against giving information, through electronic means, about how to die. That means that a website giving information about obtaining the lethal drug Nembutal and how much to take for a peaceful death is illegal. It is also illegal to offer this sort of information over the telephone. This law is aimed at the organisation Exit International, which specialises in offering end-of-life information. Philip Nitschke, the director of Exit, and his partner Fiona Stewart wrote a book, The Peaceful Pill Handbook, giving detailed information about various ways to die (Nitschke and Stewart 2006). It is banned in Australia.

The Australian federal government proposed introducing compulsory Internet filtering. Ostensibly aimed primarily at paedophilia websites, the target list of websites included euthanasia sites (Duffy 2009;Electronic Frontiers Australia 2010).

Nitschke runs workshops about end-of-life options. In Australia, he is restricted about what he can say in a public meeting, so for more detailed information — such as that given in The Peaceful Pill Handbook — the meeting is closed and everyone in attendance has to join Exit International and, to prevent prosecution, sign a disclaimer stating that “none of the information provided in this workshop will be used in any way to advise, counsel or assist in the act of suicide, either my own, or any other persons.” Nitschke has run the same workshops in other countries, including Britain, Canada, New Zealand and the US. In several cities, meetings have had to be cancelled or moved at the last moment after the organisation providing a venue — a church or library, for example — has withdrawn permission to use its facilities. Sometimes this withdrawal of permission has been despite constitutional free-speech guarantees such as found in Canada and the US (Bermingham 2009).

### Devaluation

People desiring to end their life often are assumed to be depressed (Jeffrey 2009, p. 81) or experiencing “demoralization syndrome” (Kissane et al. 2000), namely suffering a mental illness. Some of those who assist others to die have been denigrated. For example, the US media dubbed right-to-die activist Jack Kevorkian “Dr. Death” and subjected him to “caustic character assaults” (Gailey 2003, p. 190).

### Reinterpretation

Some critics say euthanasia is not appropriate because suffering from pain, breathlessness and nausea can be alleviated by well-resourced palliative care (Jeffrey 2009). Such claims may downplay the suffering associated with incapacity and loss of autonomy or due to less-than-effective palliative care. Reinterpretation by blaming can occur when a person’s desire to die is attributed to beliefs considered by some to be inappropriate. For example, some people believe they are a burden to their families — a belief sometimes fostered by family members — and hence it might be better if they were dead; reinterpretation by blaming can occur when such motivations are incorrectly attributed to individuals. Probably the most important reinterpretation is framing: seeing life as inherently valuable, irrespective of suffering.

### Official channels

In most countries, where euthanasia is illegal, appealing to medical or legal authorities to hasten one’s death is futile: official channels endorse the denial of euthanasia rather than providing relief from it. Sometimes it is possible to obtain covert assistance to die from a sympathetic doctor or nurse; however, making a public request for such assistance actually makes it more difficult to obtain, because medical personnel know they will then be under greater scrutiny (Nitschke and Stewart 2006).

### Intimidation

In most countries, assisting someone to die is illegal. One possible outcome is being charged with murder; Jack Kevorkian was brought to trial for murder on several occasions (Nicol and Wylie 2006, pp. 184–195). Trials of individuals for assisting a death send a warning to others to avoid this risk. Even short of a trial, conviction and jail, the prospect of being investigated can cause individuals to avoid assisting a death or speaking out about their past or likely future involvement in someone’s death.

Each of these methods of reducing awareness, concern and action against the denial of euthanasia can be challenged. Some examples follow.

### Exposure

In recent years, a few individuals — such as Derek Humphry ( 2002), Jack Kevorkian (Nicol and Wylie 2006) and Philip Nitschke (Nitschke and Stewart 2005) — have dramatically increased awareness of the issue of euthanasia (Côté 2012). Their initiatives have received extensive media coverage. As individuals, they are the most public face of voluntary euthanasia campaigning.

The spread of the Internet has made censorship of options for dying far more difficult. To outflank the Australian government’s ban on electronic communication of options for dying, Exit International put its phone line in New Zealand and hosts its website outside Australia. The Peaceful Pill Handbook is available in both printed and electronic editions and can readily be purchased in Australia from foreign outlets with little risk of detection.

### Validation

Whenever a person behaves with dignity, compassion and other valued traits, their behaviour gains in stature. The most important way of countering devaluation in the euthanasia debate has been to personalise the issue.

On several occasions, individuals with terminal illnesses have spoken out in support of being able to end their lives when, where and how they choose — and spoken in a calm, rational, sensible fashion, often despite great suffering (Nitschke and Stewart 2005;pp. 256–266; Syme , pp. 256–266; Syme pp. 256–266; Syme 2008, pp. 231–244). Such individuals evoke sympathy and challenge the image of depression and irrationality.

The same applies to advocates of euthanasia. For example, Rodney Syme, a doctor based in Melbourne, spoke in public about his assistance to patients who wished to die, and gradually became more prominent in support of voluntary euthanasia. His status as a doctor, his sensitivity to suffering and his sensible discussion of the pros and cons of the issue help to validate the position he advocates (Syme 2008).

### Reframing

Advocates frame the issue in ways that emphasise what they believe are the key injustices: unnecessary suffering and the denial of the right to die. Language is important: “death with dignity” and “right to die” are ways of framing the issue that avoid the negative connotations of “euthanasia” and “suicide,” not to mention “killing” and “murder.” Some societies have changed their names from “Voluntary Euthanasia” to “Dignity in Dying” or related names to avoid connotations of the word euthanasia. The Hemlock Society in the US has become Compassion and Choices.

### Mobilisation

Supporters of voluntary euthanasia have used a wide range of methods to promote their goals, including creating organisations, holding meetings, advising individuals, publishing leaflets and articles, seeking media coverage and, in some cases, supporting those who defy the laws, in what might be called civil disobedience. These organisations and activities collectively can be said to constitute a social movement (Côté 2012;Hillyard and Dombrink 2001;McInerney 2000).

Some organisations seek legal change largely through putting pressure on governments, an approach that can be seen as using official channels. Others, for instance Exit International, advocate direct action, namely enabling individuals to obtain knowledge and tools for their own deaths (Nitschke and Stewart 2005).

### Resistance

Some supporters of voluntary euthanasia have defied laws. Jack Kevorkian developed a machine to allow people to end their lives and used it in the face of lawmakers seeking to prosecute him. (Eventually he was jailed for murder [Nicol and Wylie 2006). Philip Nitschke, in his book The Peaceful Pill Handbook and in Exit seminars, provides information to people on how they can end their lives — though some of the methods involve breaking the law. For example, many Australians have visited Mexico and legally purchased Nembutal but then illegally brought it back to Australia in case they feel the need to use it.

In summary, in countries in which euthanasia is illegal, there is an ongoing struggle between its opponents and supporters. In some countries, for example Australia, the measures taken by opponents include cover-up, devaluation, reinterpretation, official channels and intimidation — every one of the techniques typically used to reduce outrage over something seen by others as unjust.

In this analysis, I have not tried to assess whether refusing people’s requests to die actually is an injustice. The analysis here is of tactics. It is possible to say that some of the tactics used by opponents of people’s requests to die are characteristic of acting in ways that reduce outrage over injustice.

## Conclusion

In many studies of euthanasia, the focus is on medical, legal or moral dimensions of the issue. Here a different focus is introduced: on tactics used to reduce or increase outrage. This analysis is based on the observation that the same types of tactics are used in a wide range of areas — from sexual harassment to genocide — by powerful perpetrators to reduce public discontent about something perceived as wrong. Powerful perpetrators — especially when backed by government — can use the techniques of cover-up, devaluation, reinterpretation, official channels and intimidation. On the other hand, weak perpetrators, such as doctors who euthanise patients without official sanction and without significant resources, typically rely mostly on cover-up.

By highlighting and classifying the tactics used in a contentious area, it is possible to say whether the tactics are characteristic of a powerful perpetrator doing something potentially perceived as unfair (Martin 2007). This approach is well suited to the euthanasia debate because there are two potential injustices involved: the injustice of euthanasia and the injustice of denying euthanasia.

An examination of the infamous Nazi T4 programme reveals ample evidence that the perpetrators used all five methods to reduce outrage. The obvious inference is that the programme, in the eyes of many parents, relatives and other citizens, was highly objectionable, which is exactly what historians of the programme have documented.

The other potential injustice is denial of euthanasia. Here, also, there is evidence, at least in some places, of all five methods of reducing outrage. The implication is that many people believe that this denial is unjust — again, something that is expected, given that opinion polls reveal considerable support for euthanasia in appropriate circumstances. On the other hand, in places such as the Netherlands and Oregon where euthanasia is legal, there is little evidence of the methods for reducing outrage. Some critics are indeed outraged by the practices in these places, but authorities and supporters do not seem to have adopted methods to suppress opposition.

One value of this analysis is showing ways for challengers to increase outrage over practices they oppose. To do this, it is useful to expose the actions, validate those who are the targets, interpret the actions as unjust, mobilise support (and avoid or discredit official channels), and resist intimidation. Groups in the right-to-die movement have used most of these methods. It is noteworthy that many voluntary euthanasia groups put much of their energy into official channels, namely seeking law reform, which actually dampens outrage.

Given that two competing injustices are involved — euthanasia and its denial — analysis of tactics provides a way of judging appropriate behaviour. Those who believe in a fair treatment of the issues can ask the following sorts of questions.

- Are we open about what is happening and do we allow others to present their viewpoints without hindrance?
- Are we respectful of everyone involved?
- Are we truthful? Do we accept responsibility?
- Do we encourage as many people as possible to be involved with the issue, whatever their viewpoint?
- Do we act to reduce threats to and coercion of anyone involved?

The framework presented here for examining tactics in the euthanasia controversy can be used to examine tactics used in other controversial health issues, such as abortion and stem-cell research. The basic approach is to examine the methods used by powerful players on each side. If these fit a consistent pattern of covering up adverse information, devaluing others, reinterpreting the events using lying, blaming and minimising, using official channels to give an appearance of justice without the substance, and intimidating opponents, this is characteristic of the behaviour of powerful perpetrators of injustice. If, on the other hand, the tactics fit a consistent pattern of being open about information, respecting everyone including opponents, avoiding deception and blaming, not relying on authoritative endorsement, and avoiding intimidation, this is characteristic of an approach that is open and honest.

A consistent pattern of tactics does not indicate whether a side is right or ethical, but it does suggest whether or not it is “playing fair” and allowing audiences to obtain information, study arguments and assess the issue free of deception, threats and inducements. The normal approach to health controversies is to assess the issues to decide which arguments are correct or which actions are appropriate; actions to support one’s preferred stance may not be studied or questioned, whereas unsavoury ones taken by opponents are castigated. By looking for patterns in tactics, a different perspective is provided on the controversy. Sometimes actions are more revealing than words.
